# Stringent Selection of Knobby *Plasmodium falciparum*-Infected Erythrocytes during Cytoadhesion at Febrile Temperature

**DOI:** 10.3390/microorganisms8020174

**Published:** 2020-01-25

**Authors:** Michael Dörpinghaus, Finn Fürstenwerth, Lisa K. Roth, Philip Bouws, Maximilian Rakotonirinalalao, Vincent Jordan, Michaela Sauer, Torben Rehn, Eva Pansegrau, Katharina Höhn, Paolo Mesén-Ramírez, Anna Bachmann, Stephan Lorenzen, Thomas Roeder, Nahla Galal Metwally, Iris Bruchhaus

**Affiliations:** 1 Bernhard Nocht Institute for Tropical Medicine, 20359 Hamburg, Germany; 2 Molecular Physiology Department, Zoological Institute, Christian-Albrechts University Kiel, 24118 Kiel, Germany

**Keywords:** Malaria, transcriptome, *Plasmodium falciparum*, CSA, CD36, ICAM-1, knobs, cytoadhesion, fever

## Abstract

Changes in the erythrocyte membrane induced by *Plasmodium falciparum* invasion allow cytoadhesion of infected erythrocytes (IEs) to the host endothelium, which can lead to severe complications. Binding to endothelial cell receptors (ECRs) is mainly mediated by members of the *P. falciparum* erythrocyte membrane protein 1 (*Pf*EMP1) family, encoded by *var* genes. Malaria infection causes several common symptoms, with fever being the most apparent. In this study, the effects of febrile conditions on cytoadhesion of predominately knobless erythrocytes infected with the laboratory isolate IT4 to chondroitin-4-sulfate A (CSA), intercellular adhesion molecule 1 (ICAM-1), and CD36 were investigated. IEs enriched for binding to CSA at 40 °C exhibited significantly increased binding capacity relative to parasites enriched at 37 °C. This interaction was due to increased *var2csa* expression and trafficking of the corresponding *Pf*EMP1 to the IE surface as well as to a selection of knobby IEs. Furthermore, the enrichment of IEs to ICAM-1 at 40 °C also led to selection of knobby IEs over knobless IEs, whereas enrichment on CD36 did not lead to a selection. In summary, these findings demonstrate that knobs are crucial for parasitic survival in the host, especially during fever episodes, and thus, that selection pressure on the formation of knobs could be controlled by the host.

## 1. Introduction

After decades of research, malaria remains a major public health burden. Malaria is a tropical disease caused by five different human pathogenic *Plasmodium* species, of which *P. falciparum* is the most deadly. During *P. falciparum* infection, a number of non-specific symptoms such as fever, headache, weakness, myalgia, chills, and dizziness occur, and in severe cases, the patient’s consciousness can be impaired. The infection can ultimately lead to seizures, coma, and death [1]. These clinical symptoms are related to the life cycle of the parasite. During its life cycle in the human host, the parasite invades erythrocytes. The infected erythrocytes (IEs) adhere to endothelial cells, which obstructs blood flow in microvessels. This can lead to hypoxia, induction of the inflammatory response, tissue damage, and organ failure [2,3].

IE cytoadhesion is mediated by variant surface antigens of the protein family *P. falciparum* erythrocyte membrane protein 1 (*Pf*EMP1) [2,4,5]. *Pf*EMP1 proteins are encoded by approximately 60 *var* genes that are exclusively expressed, meaning that at any given time, only one *Pf*EMP1 family member is present on the IE surface [6]. *Pf*EMP1 molecules can bind to various receptors on endothelial cell surfaces, such as CD36, intercellular adhesion molecule-1 (ICAM-1), endothelial protein cell receptor (EPCR), and chondroitin-4-sulfate A (CSA). Thus far, at least 23 endothelial cell receptors (ECRs) that interact with IEs have been identified [3,7,8,9,10,11,12,13]. 

According to the presence of ECRs, and thus the location of adhesive IEs, malaria infection has different clinical outcomes. IE adherence to brain endothelial cells can cause cerebral malaria, leading to neurocognitive impairment, coma, and potentially death [14,15]. If IEs adhere to CSA expressed in the placental intervillious space, pregnancy-associated malaria (PAM) can occur. High levels of cytoadhesive IEs can interfere with the transmission of vital substances through the placenta, which often leads to death of the unborn child. The *Pf*EMP1 molecule VAR2CSA was identified as a binding partner with binding preference to low-sulfated CSA located in the placenta [16,17]. However, cytoadhesion of IEs exposing VAR2CSA is not limited to CSA on placental syncytiotrophoblasts. CSA-binding isolates have also been detected in non-pregnant patients [18,19,20]. In addition, a similar modification of chondroitin sulfate is found on a high proportion of malignant cells and binds recombinant VAR2CSA [21,22].

*Pf*EMP1 proteins are concentrated and anchored to host cell actin filaments in electron-dense protrusions of the erythrocyte plasma membrane, known as knobs. Knobs consist of various submembrane structural proteins, including the predominant protein of this structure, knob-associated histidine-rich protein (KAHRP). Further, additional proteins are necessary for the formation of knobs, including *Pf*EMP3, ring-infected erythrocyte antigen (RESA), mature parasite-infected erythrocyte surface antigen (MESA)/*Pf*EMP2, and *Pf*332 [23]. At least one *Plasmodium* helical interspersed subtelomeric (PHIST) protein has been identified, which interacts with *Pf*EMP1 and co-migrates to IE knob structures [24]. Prior studies have identified that during continuous in vitro culture, *P. falciparum* frequently undergoes subtelomeric deletions in at least one of its 14 chromosomes, often in the subtelomeric region of chromosome 2, where genes encoding knob structural proteins such as *kahrp* and *pfemp3* are located [25]. Therefore, parasites in cell culture often lose the ability to form knob structures. However, these subtelomeric deletions could also be detected in field isolates [26].

Fever is an immune response of the host that can be triggered by pathogens in the course of infection. Febrile temperature affects the *Plasmodium* parasite in several ways. A study by Oakley et al. investigated the effect of elevated temperature on the parasite by analyzing gene expression using a microarray approach. Oakley et al. identified that one of the most notable transcriptional changes occurred in genes encoding proteins exported to the host cell cytoplasm [27]. Prior studies have also demonstrated that fever induces cytoadhesion of IEs to target cells, promotes the cytoadhesion of ring-stage IEs and inhibits *P. falciparum* growth. Temperatures above 41 °C lead to death of the parasite [28,29,30].

The present study sought to determine if and how febrile temperature affects cytoadhesion of IEs to CSA, ICAM-1, and CD36. In this context, we found that the immortalized brain endothelial cell line HBEC-5i is suitable as a model to study cytoadherence of IEs to CSA. By enriching parasites to CSA on the surface of HBEC-5i cells at 40 °C, we identified that the binding capacity of these IEs to CSA is enhanced compared with IEs enriched at 37 °C. This correlated with increased IE *var2csa* expression and thus increased cell surface VAR2CSA in IEs at febrile temperature. Moreover, at 40 °C, knobby IEs were enriched, while knobless IEs dominated the population enriched at 37 °C. However, heat shock alone did not lead to selection of knobby IEs. Furthermore, enrichment of IEs on ICAM-1 at 40 °C also led to the selection of knobby IEs, while in the population enriched on CD36 at 40 °C, knobless IEs dominated. Transferred to in vivo conditions, this may mean that at febrile temperatures the binding of IEs to certain receptors is more stable when knobs are present. Thus, knobs are crucial for parasitic survival in the host, especially during fever episodes. 

## 2. Materials and Methods 

### 2.1. Parasite and Eukaryotic Cell Culture

*P. falciparum* isolate IT4 (FCR3S1.2) was cultivated using human O^+^ erythrocytes (5% hematocrit; UKE, Hamburg, Germany) in RPMI 1640 medium (AppliChem, Darmstadt, Germany) containing 10% human serum A^+^ (Interstate Blood Bank Inc., Memphis, TN, USA) according to standard procedures [31]. Parasites were synchronized at least every 2 weeks using 5% sorbitol solution [32]. Human HBEC-5i brain endothelial cells (American Type Culture Collection (ATCC), Manassas, VA, USA; no. CRL-3245) were seeded in 0.1% gelatin-coated T25 culture flasks. For normal cell culture, DMEM/F-12 complete growth medium (Gibco, Thermo Fisher Scientific, Bremen, Germany) containing 40 µg/mL endothelial cell growth supplement (ECGS; Merck Millipore, Darmstadt, Germany), 10% heat-inactivated fetal calf serum (Capricorn Scientific, Ebsdorfergrund, Germany), and 0.1 mg/mL gentamycin (Sigma–Aldrich Merck, Darmstadt, Germany) was used. Transfected CHO-745 cells were cultivated in Ham’s F12 medium (Capricorn Scientific, Ebsdorfergrund, Germany) supplemented with 10% heat-inactivated fetal calf serum (Capricorn Scientific, Ebsdorfergrund, Germany) and penicillin-streptomycin (0.1 U/mL; Gibco, Thermo Fisher Scientific, Bremen, Germany). Transfection of CHO-745 cells was performed as described previously [33]. G418 (0.7 mg/mL; Geneticin; Thermo Fischer Scientific) was used as a selection marker for transfected cells. Cell and parasite cultures used for the study were verified to be free of mycoplasma.

### 2.2. Enrichment of HBEC-5i, CD36, and ICAM-1 Binding Parasites

To enrich IEs binding to HBEC-5i cells, CD36, or ICAM-1, synchronized trophozoite-stage IEs (at least 5% parasitemia, 1% hematocrit) were suspended in binding medium (RPMI 1640 medium supplemented with 2% glucose, pH 7.2; Gibco, Thermo Fisher Scientific, Bremen, Germany). Subsequently, IEs were added to a monolayer (50–90% confluence) of HBEC-5i cells (EC) or transgenic CHO-745 cells expressing ICAM-1 (CHO^ICAM-1^) or CD36 (CHO^CD36^) on the surface [33]. Subsequently, cells were co-incubated at 37 °C (5% CO_2_) for 60 min with gentle orbital shaking every 15 min in all directions. To select for IEs that bound to heat-shocked HBEC-5i cells (EC40°), HBEC-5i cells were pre-incubated at 40 °C (5% CO_2_) for 7 h and co-incubated with IEs at 40 °C for another 60 min. To select parasites bound to heat-shocked CHO^ICAM-1^ and CHO^CD36^ cells, CHO cells were pre-incubated at 40 °C for 4 h, before addition of parasites for another 60 min at 40 °C. After co-incubation, non-bound IEs were removed by washing five to eight times with binding medium. Cytoadhesion was confirmed under an inverted microscope. To ensure growth of selected parasites, CHO^ICAM-1^, CHO^CD36^, or HBEC-5i cells and bound IEs were cultivated for 24 h following *P. falciparum* standard procedure. Ring-stage parasites were harvested on the following day, and any remaining CHO^ICAM-1^, CHO^CD36^, or HBEC-5i cells were removed using Biocoll separation solution (Biochrom, Berlin, Germany). The selected IEs were cultured to parasitemia of at least 5%, and the entire process was repeated. The resulting *P. falciparum* populations enriched at 37 °C or 40 °C were termed IT4^CSA^EC37° or IT4^CSA^EC40° (enriched to HBEC-5i cells), IT4^ICAM-1^CHO^ICAM-1^37° and IT4^ICAM-1^CHO^ICAM-1^40° (enriched to CHO^ICAM-1^ cells), and IT4^CD36^CHO^CD36^37° and IT4^CD36^CHO^CD36^40° (enriched to CHO^CD36^ cells). As a control, *P. falciparum* cultivated under standard conditions (IT4^NE^, non-enriched) was used for all further experiments.

### 2.3. Static Binding Assay and CSA Inhibition Assay

A static binding assay was performed to verify the binding capacity of enriched parasites. Seventy-two h before the assay, parasites were synchronized. On the same day, HBEC-5i and CHO^CD36^ cells were seeded on 0.1% gelatin-coated 13 mm coverslips at a density of 1.2 × 10^6^ cells/mL. On the day of the assay, confluence was assessed with an inverted microscope. Ideally, the cells formed a monolayer. For inhibition assays, HBEC-5i and CHO^CD36^ cells were pre-incubated with 10 µg/mL soluble CSA (sCSA; Sigma–Aldrich Merck, Darmstadt, Germany) at 37 °C for 30 min. Trophozoite-stage parasites (5% parasitemia, 1% hematocrit) were resuspended in binding medium, and the suspension was added to the cell monolayers and incubated for 1 h at 37 °C or 40 °C under 5% CO_2_ with gentle orbital shaking every 15 min. Subsequently, coverslips were gently washed with binding medium to remove non-bound IEs. The remaining IEs were fixed with 1% glutaraldehyde in phosphate-buffered saline (PBS; 0.14 M NaCl, 0.3 mM KCl, 8 mM NaH_2_PO_4_, 1.5 mM KH_2_PO_4_, pH 7.4) for 30 min at room temperature (RT). Fixed cells were stained with a filtered Giemsa/Weisser buffer solution (1:10) for another 30 min. The number of adherent IEs was determined by counting the number of bound IEs on 300 HBEC-5i, CHO^CD36^, or CHO^ICAM-1^ cells under a light microscope. Assays were performed at least two times in triplicate.

### 2.4. DNA and RNA Purification, Library Preparation, and Transcriptome Analysis

Total DNA was isolated using the QIAamp DNA Mini Kit (Qiagen, Hilden, Germany) according to the manufacturer’s instructions. For RNA isolation, parasites were synchronized 48 h prior to harvest. Ring-stage IEs were rapidly lysed in a 10-times higher volume of pre-warmed 37 °C TRIzol (Invitrogen, Thermo Fisher Scientific, Bremen, Germany) followed and incubated for 5 min at 37 °C. Subsequently, the samples were stored at -80 °C. RNA was isolated using a PureLink RNA Mini Kit (Thermo Fisher Scientific, Bremen, Germany) according to the manufacturer’s instructions. Genomic DNA contamination was removed using the TURBO DNA-free Kit (Invitrogen, Thermo Fisher Scientific, Bremen, Germany) followed by a magnetic bead enzymatic wash using Agencourt RNAClean XP (Beckman Coulter, Krefeld, Germany). The concentration and quality of isolated RNA were assessed using an Agilent 2100 Bioanalyser System with the Agilent RNA 6000 Pico Kit (Agilent Technologies, Ratlingen, Germany). The RNA was sent to BGI (Shenzhen, China), where RNAseq was performed using the Illumina HiSeq 4000 PE100 platform (approximately 11 M PE reads per samples). Reads were trimmed and filtered using Trimmomatic [34], and aligned to IT4 genome data available at PlasmoDB [35] using RSEM [36] and Bowtie2 [37] software. Differential expression was tested using DEseq for normalization of the row reads [38]. P-values were adjusted using Holm’s method.

### 2.5. Trypsin Assay and Western Blot

To measure surface-exposed *Pf*EMP1, a trypsin cleavage assay was performed using synchronized trophozoite-stage parasites (24–28 h after erythrocyte infection). IEs were isolated with magnetic cell sorting (CS columns; Miltenyi Biotec, Bergisch Gladbach, Deutschland). Subsequently, the cell count was set to 1 × 10^6^ cells/µL and split into two fractions, with and without addition of 1 µg/mL trypsin for 30 min at 37 °C. Both fractions were resuspended in 10 mM HEPES buffer (pH 7.2) and lysed using three freeze–thaw cycles in liquid nitrogen. The supernatant and pellet were separated by centrifugation at 20,000× *g* for 10 min at 4 °C. The supernatant was removed, and the pellet containing the membrane fraction was washed twice with PBS. For western blotting, cell membranes were lysed in 2× Laemmli buffer and heated for 5 min at 95 °C. An equivalent of 1 × 10^7^ cells/lane were separated on a 6% SDS gel at 400 mA and blotted onto nitrocellulose membranes. Uniform loading of the gels was confirmed by staining a second gel in parallel with Coomassie Blue. Subsequently, membranes were blocked with 5% milk in TBS (0.3 M NaCl, 20 mM Tris pure, pH 7.5) for 30 min. Primary antibodies were diluted in 2.5% dry milk/TBS as follows: mouse anti-ATS-GHI-monoclonal 1:500 (WEHI Antibody Facility, Walter and Eliza Hall Institute of Medical Research, Melbourne, Australia); rabbit anti-spectrin, 1:2000 (Sigma–Aldrich Merck, Darmstadt, Germany); rabbit anti-KAHRP, 1:4000 (a kind gift from Prof. Brian Cooke, Monash University, Melbourne, Australia). Membranes were incubated with primary antibodies overnight at 4 °C. The secondary antibodies were diluted in 5% dry milk/TBS as follows: rabbit anti-mouse 1:5,000 (Agilent Dako, Santa Clara, CA, USA); porcine anti-rabbit 1:10,000 for spectrin and 1:20,000 for KAHRP blots (Agilent Dako, Santa Clara, CA, USA). Membranes were incubated with secondary antibodies for 2 h at RT. The chemiluminescent signal of the HRP-coupled secondary antibodies was visualized on an Amersham Hyperfilm-ECL (GE Healthcare, Freiburg, Germany). Densitometric quantification was performed with ImageJ 1.52a and Adobe Photoshop CS3 Extended (version 10.0.1).

### 2.6. Electron Microscopy

Synchronized trophozoite-stage *P. falciparum* cultures 24–28 h post-infection of erythrocytes were separated using 80%, 60%, and 40% percoll density gradient centrifugation, and fixed with 2.5% glutaraldehyde/2.5% paraformaldehyde (Electron Microscopy Sciences, München, Germany). Samples were washed with 50 mM cacodylate buffer, pH 7.4 (Electron Microscopy Sciences, München, Germany), and post-fixed with 2% OsO_4_ for 45–60 min on ice in darkness. After heavy-metal staining with 0.5–1% uranylacetate (Electron Microscopy Sciences, München Germany) for 45–60 min at RT, samples were dehydrated with an increasing ethanol series. Following embedding in epoxy resin (EPON) (Carl Roth, Karlsruhe, Germany), the polymerized samples were cut with an Ultracut UC7 (Leica, Wetzlar, Germany) in 55–60 nm thick sections and collected on 300 mesh copper grids (Plano, Wetzlar, Germany). Sections were analyzed with a transmission electron microscope (Tecnai Spirit, FEI Company, Hillsboro, OR, USA) at an accelerating voltage of 80 kV.

### 2.7. Quantitative Real-Time PCR

For qPCR experiments, sense and antisense primers were designed to amplify 100–120 bp fragments of the target genes. The oligonucleotides used for PCR are listed in Table S1. After RNA isolation, cDNA was synthesized using SuperScript II Reverse Transcriptase (Invitrogen, Thermo Fisher Scientific, Bremen, Germany) and random hexamers (Invitrogen) at 50 °C for 1 h. The cDNA/gDNA template was mixed with SYBR Green PCR Master Mix (QuantiTect SYBR Green PCR Systems; Qiagen, Hilden, Germany) and 0.5 µM forward and reverse primer at a final volume of 10 µl. Reactions were incubated at 95 °C for 15 min, and then subjected to 40 cycles of 95 °C for 15 s and 60 °C for 1 min, and a subsequent melting step (60–95 °C). The specificity of each primer pair was confirmed after each qPCR run using melt curve analysis. The conserved ring-stage expressed gene *sbp1 (skeleton-binding protein 1)*, and the housekeeping genes *fructose-bisphosphate aldolase* and *arginyl-tRNA synthetase* were used to normalize *var* gene expression (as a control, a single RNA biological sample isolated from IT4^CSA^EC37° and IT4^CSA^EC40° was used to verify qPCR results). To compare *var* gene expression levels in each sample, relative gene expression was calculated for each individual *var* gene relative to the geometric mean of the three normalizers after calculation of primer efficiency [39,40]. Statistical significance was tested using the Wilcoxon rank sum test (GraphPad Prism 7). 

## 3. Results

### 3.1. Cytoadhesion of Infected Erythrocytes to HBEC-5i Cells Was Mediated by VAR2CSA

**Figure 1:**
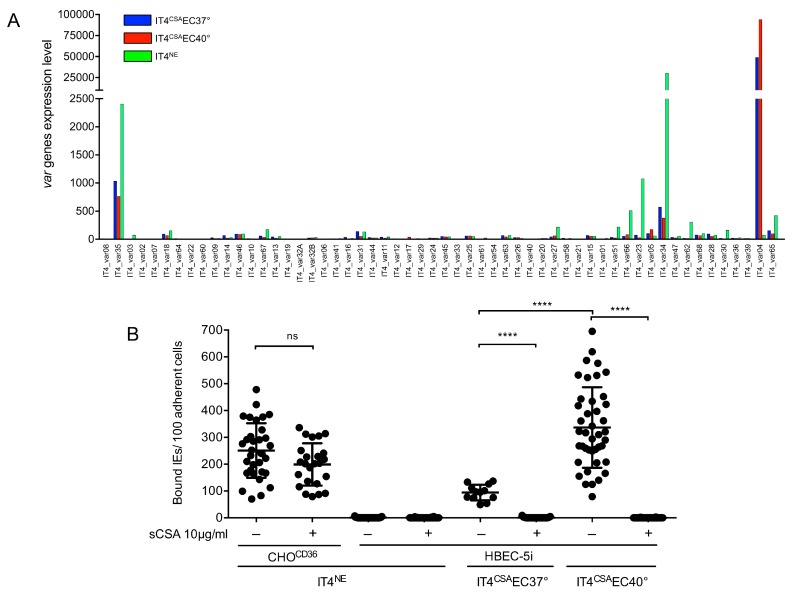
*Var* expression profile of IT4 populations enriched for binding to HBEC-5i cells at 37 °C and 40 °C, and binding capacity of different IT4 populations to CHO^CD36^ and HBEC-5i cells. (**A**) Expression profile of all *var* genes in IT4 populations enriched for binding HBEC-5i cells at 37 °C and 40 °C (IT4^CSA^EC37° and IT4^CSA^EC40°) in comparison with long-term cultured IT4 parasites (IT4^NE^). The expression level is defined as the average of normalized read counts of four independent transcriptional analyses. (**B**) Binding capacity of IT4^NE^ to transgenic CHO-475 cells expressing CD36 (CHO^CD36^) and HBEC-5i cells, and of IT4 parasites enriched for binding to HBEC-5i cells at 37 °C and 40 °C (IT4^CSA^EC37°, IT4^CSA^EC40°). Preincubations of IEs without (−) or with (+) 10 µg/mL sCSA is indicated. Each point represents one binding assay. IT4^NE^/CHO^CD36^: (-) n = 33, (+) n=24; IT4^NE^/HBEC-5i: (−) n = 42, (+) n = 32; IT4^CSA^EC37°/HBEC-5i: (−) n = 12, (+) n = 29; IT4^CSA^EC40°/HBEC-5i: (−) n = 27, (+) n = 36. Statistical significance was assessed by ordinary one-way ANOVA and Tukey’s post hoc test (^ns^ *p* > 0.05; **** *p* < 0.0001).

After long-term cultivation of *P. falciparum* IT4 isolate, IEs were rarely capable of adherence to HBEC-5i cells. To identify the effects of febrile temperature on cytoadhesion of *P. falciparum* to HBEC-5i cells, the IT4 isolate (IT4^NE^; non-enriched) was enriched for binding to HBEC-5i cells at 37 °C and 40 °C. After 5–7 consecutive rounds of enrichment, saturation of the binding capacity was observed. To identify the *var* genes expressed specifically in the HBEC-5i-enriched populations, the transcriptome profiles of the parasite populations were determined by RNAseq and evaluated, focusing on *var* gene expression in ring-stage parasites (6–10 h post-infection) (Figure 1A and Tables S2–S5). The IT4^NE^ parasite population expressed a mixture of *var* genes. Most parasites expressed group C *IT4_var34* (79%) and group A *IT4_var35* (6%), followed by *IT4_var23* (3%) and *IT4_var59* (2%). All other *var* genes were sparsely expressed, representing 1.5% or less of all *var* transcripts. IT4 parasites enriched for HBEC-5i binding significantly overexpressed group E *IT4_var04*, also known as *var2csa* (Figure 1A and Tables S2–S5). At physiological as well as febrile temperatures, expression of *var2csa* was dominant (representing 93% and 97% of all *var* transcripts, respectively). While both parasite populations only expressed *var2csa*, the number of transcripts for *var2csa* nearly doubled at 40 °C (relative read count, 94134) compared with 37 °C (relative read count, 49285) (Figure 1A and Table S2). This result could also be confirmed with the help of qPCR (Table 1). CSA is the binding partner of VAR2CSA [16,17]. To determine whether the interaction partner on the HBEC-5i cells was, in fact, CSA or another receptor, a static cytoadhesion assay was performed using trophozoite-stage IEs and HBEC-5i cells in the absence and presence of soluble CSA (sCSA) at 37 °C. sCSA did not block binding of IEs to HBEC-5i cells because IT4^NE^ parasites capable of binding to CD36 adhered at similar levels when pretreated with sCSA (250 ± 101 and 199 ± 79 IEs/100 CHO^CD36^ cells) (Figure 1B). However, pre-incubation of physiological and febrile temperature HEBC-5i-enriched parasite populations with sCSA completely abolished cytoadherence to HBEC-5i cells. Interestingly, the binding capacity of IT4 parasites enriched for binding to HEBC-5i cells at 40 °C (338 ± 150 bound IEs/100 HBEC-5i cells) was significantly increased compared with the binding capacity at 37 °C (70 ± 44 bound IEs/100 HBEC-5i) (Figure 1B). Because CSA is the binding partner of IEs on HEBC-5i cells, the HEBC-5i enriched parasite populations are subsequently referred to as IT4^CSA^EC37° (enriched to HBEC-5i cells at 37 °C) and IT4^CSA^EC40° (enriched to HBEC-5i cells at 40 °C).

**Figure 2:**
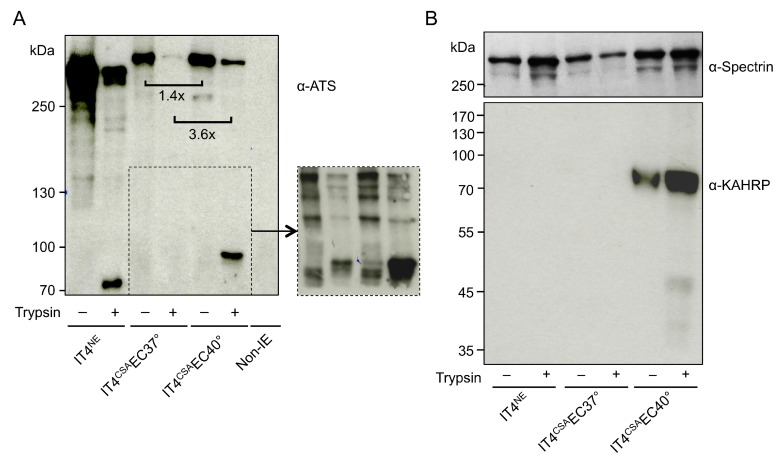
Infected erythrocyte (IE) surface *P. falciparum* erythrocyte membrane protein 1 (*Pf*EMP1) presentation, and knob-associated histidine-rich protein (KAHRP) expression of IT4 populations enriched for binding to HBEC-5i cells at 37 °C and 40 °C. (**A**) Western blot analysis for *Pf*EMP1 presentation was performed on trypsin-treated or untreated IEs and uninfected erythrocytes using an antibody against the conserved intracellular amino-terminal sequence (ATS) domain of *Pf*EMP1 molecules (α-ATS). All samples were solubilized in Laemmli sample buffer, separated by 6% SDS-PAGE and analyzed by immunoblotting. Equivalents of 1 × 10^7^ cells were loaded in each lane. The cutout represents a Western Blot with 3-times higher parasite lysate load (3 × 10^7^ IEs; Figure S1). Comparison of the relative amounts of protein was normalized to total protein amount using a corresponding Coomassie-stained gel (Figure S2). (**B**) Western blotting for KAHRP was performed with the IE membrane fraction, using α-KAHRP and α-spectrin as controls.

To determine if increased *var2csa* expression in IT4^CSA^EC40° relative to IT4^CSA^EC37° was solely due to the temperature increase, IT4^NE^ parasites were incubated once weekly (over 5 weeks) at 38.5 °C and 40 °C for 2 h without contact with HBEC-5i cells. Neither *var2csa*-specific upregulation nor upregulation of other selected *var* genes was observed (Table 1). A trypsin cleavage assay revealed that increased *var2csa* expression correlated with increased protein quantity of VAR2CSA (Figure 2A). The extracellular domain of *Pf*EMP1 molecules can be cleaved using trypsin, and the remaining intracellular acidic terminal segment (ATS) region (70–90 kDa) can be detected by western blot using an ATS-specific antibody. The amount of intracellular *Pf*EMP1 was 1.4- (no trypsin treatment) and 3.6- (with trypsin treatment) times higher in IT4^CSA^EC40° relative to IT4^CSA^EC37°. The IT4^CSA^EC40° population exhibited a 90 kDa protein band after trypsin digestion, but no protein could be detected in the IT4^CSA^EC37° population. To determine if surface *Pf*EMP1 was present at any level in the 37 °C enriched population, another western blot with 3-times higher parasite lysate load (3 × 10^7^ IEs) was performed, revealing that IT4^CSA^EC37° exhibited detectable surface *Pf*EMP1 (Figure 2A and Figure S1). In the sample of IT4^NE^ parasites containing a mixture of several *Pf*EMP1s, a 70 kDa protein band was detected after trypsin digestion. The reasons for the formation of a 90 kDa fragment after cleavage of VAR2CSA from the IT4^CSA^EC37° and IT4^CSA^EC40° populations, and that of a 70 kDa fragment after cleavage of *Pf*EMP1 from the whole IT4^NE^ population (Figure 2A) remain unknown.

### 3.2. IE Enrichment to HBEC-5i Cells at Elevated Temperature Led to Strong Selection of Knobby Infected Erythrocytes

**Figure 3:**
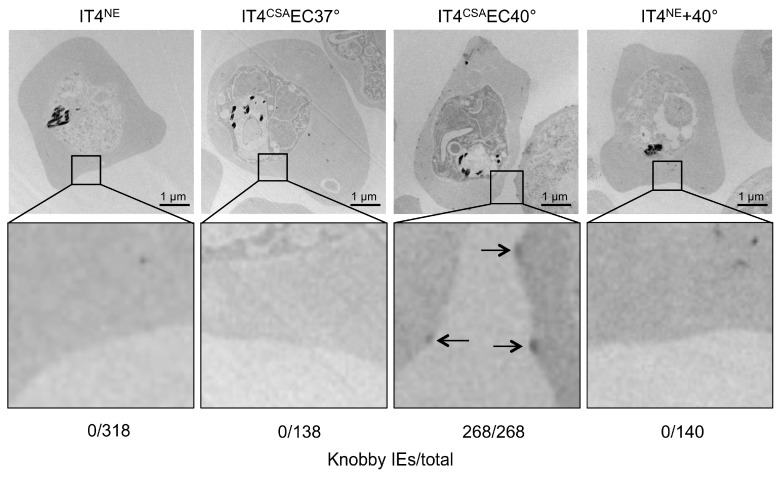
Knob formation of IT4 populations enriched for binding to HBEC-5i cells at 37 °C and 40 °C. Electron micrographs of erythrocytes infected with IT4^NE^, IT4^CSA^EC37°, IT4^CSA^EC40°, and IT4^NE^ heat-shocked at 40 °C for 2 h weekly over a period of 5 weeks. Arrows indicate knobs.

To determine if other factors in addition to the *var* gene expression and amount of VAR2CSA promoted IE binding capacity, the transcriptome profiles of ring-stage IT4^CSA^EC37° and IT4^CSA^EC40° were investigated (Tables S3–S5). In total, seven of the 17 genes that expressed 5-fold or more in IT4^CSA^EC40° relative to IT4^CSA^EC37° were known to be associated with knob formation (Table 2). Of these seven genes, four genes are located in the subtelomeric region of chromosome 2, including *kahrp*, which codes for the main component of knobs, KAHRP. Increased *kahrp* expression was verified at the protein level. KAHRP could only be detected in IT4^CSA^EC40° parasite lysates, while all other lysates were KAHRP-negative (Figure 2B). To further determine whether KAHRP was associated with the presence of knobs in the IT4^CSA^EC40° population, electron microscopy was performed (Figure 3). In accordance with the transcriptome data and protein level observations, all non-enriched (IT4^NE^, *n* = 318) and all 37 °C-enriched (IT4^CSA^EC37°, *n* = 138) parasites exhibited no obvious aggregation of electron-dense material close to the IE surface. By contrast, electron-dense truncation was observed in the parasite population enriched at 40 °C (IT4^CSA^EC40°). All 268 quantified IEs from this treatment group exhibited knobs. To determine if occurrence of knobby IEs was promoted by heat shock alone, or whether a combination of heat shock and close proximity to HBEC-5i cells was essential, IT4^NE^ parasites were heat-shocked at 38.5 °C and 40 °C for 2 h weekly over a period of 5 weeks, and subsequently analyzed by electron microscopy. No knob-like formations were observed, indicating that a combination of heat shock and co-incubation with HBEC-5i cells was required to obtain knobby IEs (Figure 3). These results were also confirmed at the RNA level. For *kahrp* and *pfemp3*, as well as for five *var* genes including *var2csa*, no increase in expression was observed in the heat-shocked IT4^NE^ population relative to control (Table 1). To determine if cytoadherence induced knob formation at febrile temperatures, or whether the enrichment process at 40 °C led to selection of knobby IEs that could be present in very small numbers in the original parasite culture, the normalized copy numbers of *kahrp* and *pfemp3* in IT4^CSA^EC37° and IT4^CSA^EC40° were determined. In IT4^CSA^EC37° parasites, the copy number of both genes was slightly increased (3.9-fold for *kahrp* and 3.3-fold for *pfemp3*) relative to IT4^NE^. However, a strong increase in copy number was observed in the IT4^CSA^EC40° population, with a 330-fold upregulation of *kahrp* and a 99-fold upregulation of *pfemp3* relative to IT4^NE^ (Table 1). Taken together, these results demonstrated that during the enrichment process on CSA at febrile temperature, knobby IEs are selected over knobless IEs.

### 3.3. Enrichment of IEs on CHO^ICAM-1^ but not CHO^CD36^Cells at Febrile Temperature Led to Selection of Knobby Infected Erythrocytes

**Figure 4:**
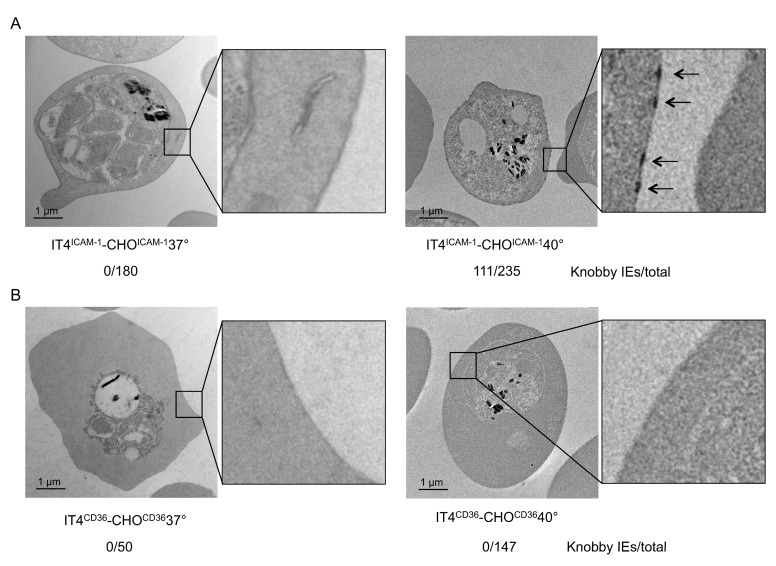
Electron micrographs of IEs enriched for binding to (**A**) CHO^ICAM-1^ at 37 °C (IT4^ICAM-1^CHO^ICAM-1^37°) and 40 °C (IT4^ICAM-1^CHOO^ICAM-1^40°), and to (**B**) CHO^CD36^ at 37 °C (IT4^CD36^CHO^CD36^37°) and 40 °C (IT4^CD36^CHOO^CD36^40°). Number of knobby IEs relative to total number of analyzed IEs is indicated.

We further verified if the selection of knobby IEs was restricted only to the VAR2CSA–CSA interaction. IT4^NE^ parasites were enriched at 37 °C and 40 °C on transgenic CHO-745 cells that exposed ICAM-1 or CD36 on their surface (CHO^ICAM-1^, CHO^CD36^). In accordance with the results of IE enrichment to CSA at 40 °C, enrichment of IEs on ICAM-1 led to selection of knobby IEs (IT4^ICAM-1^CHO^ICAM-1^40°). Knobs were detected in 47% (*n* = 235) of the IT4^ICAM-1^CHO^ICAM-1^40° population (Figure 4A). On the other hand, enrichment of IEs to CD36 at 40 °C did not lead to selection of knobby IEs (Figure 4B). These results could also be confirmed by measuring expression levels of *kahrp* and *pfemp3*. Only in the IT4^ICAM-1^CHO^ICAM-1^40° population, and not in IT4^CD36^CHO^CD36^40°, was a significant increase in expression (241-fold for *kahrp* and 56-fold for *pfemp3*) relative to control (Table 3). Increased expression of *var01* and *var16* indicated that the IT4^ICAM-1^CHO^ICAM-1^40° population, was specifically enriched to ICAM-1, as VAR01 and VAR16 are known binding partners for ICAM-1 (Table 3) [33].

## 4. Discussion

A common symptom of a malaria infection is fever, but most studies of cytoadhesion have been performed at normal body temperature. The present study revealed the impact of febrile temperature on the binding phenotype of *P. falciparum* IEs to CSA on immortalized HBEC-5i human brain endothelial cells, as well as ICAM-1 and CD36 exposed on transgenic CHO cells.

HBEC-5i cells were derived from a human cerebral cortex and, therefore, exhibited major features of cerebral endothelial cells. Prior studies established that in addition to several receptors such as ICAM-1, VCAM-1, and VE-cadherin, CSA can also be detected on the surface of HEBC-5i cells (ATCC^®^ CRL-3245^™^). The present study demonstrated that enrichment of IEs on HEBC-5i cells produced a parasite population mainly expressing *var2csa*, which encodes VAR2CSA, the binding partner of CSA [7,8,47]. Binding of the enriched parasite population to HEBC-5i cells was abrogated in the presence of sCSA, demonstrating that in this context, CSA, and not another receptor, was the binding partner for CSA. These findings implied that CSA was dominant on HEBC-5i cells, and that binding of IEs to other receptors such as ICAM-1 was shielded. Endothelial cells have been demonstrated to expose high levels of CSA in prior studies [48,49]. In this context, it is also likely that the properties of CSA-mediated cytoadhesion are consistent with the initiation of obstruction in microvessels of the internal organs [48]. Additionally, VAR2CSA binds different types of tumor cells and tissues of epithelial, mesenchymal, and hematopoietic origin, which also expose CSA on their surface [21,22]. In contrast to the results described in the present study, Claessens et al. identified *it4_var07* and *it4_var19* as the primarily expressed *var* genes after enrichment of IEs to HEBC-5i cells [50]. Why enrichment to the same immortalized cell line led to different results is unclear at present. Different culture conditions could potentially influence expression of genes encoding different receptors. In connection with our study, the HBEC-5i cells are a suitable model to investigate the interaction of IEs with CSA. Interestingly, parasites enriched to HEBC-5i cells at 40 °C (IT4^CSA^EC40°) exhibited a 2-fold upregulation of *var2csa* relative to parasites enriched at 37 °C (IT4^CSA^EC37°), resulting in increased cell surface *Pf*EMP1 protein levels. The change in *var* gene expression was not solely due to heat shock, as *var* upregulation did not occur when parasites were only exposed to febrile temperature in the absence of HEBC-5i cells. In addition, approximately 5-times as many IEs of the IT4^CSA^EC40° population bound to the HBEC-5i cells compared with the IT4^CSA^EC37° population. These results are in accordance with previous studies, which identified heat-induced enhancement of binding capacity [28,30]. However, it is unlikely that the increased expression has such a strong influence on the binding capacity.

In addition to the amount of *Pf*EMP1 on IE surfaces, additional factors can affect the interaction of IEs with ECRs. *Pf*EMP1 proteins are anchored to parasitic structures known as knobs. Knobs are submembranous protrusions that function as tight junctions between IEs and endothelial cells [51]. Numerous studies identified KAHRP, *Pf*EMP3, *Pf*EMP2 (MESA), ring-infected erythrocyte antigen (RESA), and *Pf*332 as important proteins for knob formation [23,52,53]. Alampalli et al. identified new knob constituents, including elongation factor 1 alpha, acyl-CoA synthetase, and some hypothetical proteins [46]. In addition, as mentioned above, members of the PHIST protein family also play crucial roles in binding *Pf*EMP1 co-migrating to knob structures [24]. Numerous genes important for knob formation, such as *kahrp* and *pfemp3*, are localized to the subtelomeric region of chromosome 2. Transcriptomic analysis of IT4^CSA^EC40° and IT4^CSA^EC37° parasites revealed that six genes located in the subtelomeric region of chromosome 2 are expressed in IT4^CSA^EC40° but not IT4^CSA^EC37° parasites, four of which are known to be associated with knob formation. In addition to *kahrp* and *pfemp3*, *knob-associated heat shock protein 4* and *PHISTb domain-containing RESA-like protein* 1 are also present in this region [43,44]. The other differentially expressed genes within this locus are *DnaJ*, a putative chaperone protein, and a *Plasmodium* exported protein of unknown function. Referring to Alampalli et al., in *P. falciparum*, isolate 3D7 and acyl-CoA synthetase, a glycophorin binding protein, and several PHIST-proteins were also upregulated [46]. However, it is not clear if these genes were homologous to the genes discovered in the IT4 isolate in this study. Nevertheless, this transcriptional data provides strong supporting evidence that knob formation occurred in IT4^CSA^EC40° but not in IT4^CSA^EC37° parasites.

Because KAHRP is essential for knob formation [54], we next sought to verify the transcriptional *kahrp* expression data on the protein level. KAHRP protein could only be detected in IT4^CSA^EC40° parasites, consistent with the transcriptional data. Electron microscopic analysis revealed that the presence of KAHRP protein correlated with knob formation. All quantified IT4^NE^ and IT4^CSA^EC37° IEs were knobless, while all quantified IT4^CSA^EC40° IEs exhibited knob structures at the erythrocyte membrane.

Deletion of the subtelomeric region of chromosome 2 often occurs during long-term cultivation, but was also described for patient isolates [26]. Ruangjirachuporn and colleagues examined 60 freshly isolated *P. falciparum* isolates from Gambian children for the presence of knobs on the surface of IEs. They detected knobby IE in all isolates. They also found knobless IEs in 42% of the isolates tested. However, these only account for a small proportion. Out of 616 IEs examined, only 39 were knobless, while 572 had knobs. As far as we know, it is not yet clear how frequent subtelomeric deletions occur in vivo and whether and how completely the knobless IEs are sorted out by the spleen [55]. In our study was initially unclear whether knob formation was induced by the interplay of fever and cytoadhesion, or whether knob formation resulted from the selection of knobby IEs from a mixed culture consisting mainly of knobless parasites, but with a minority population of knob-forming parasites. However, the copy numbers of *kahrp* and *pfemp3* were significantly increased in IT4^CSA^EC40° but not in IT4^CSA^EC37° compared with IT4^NE^. This leads to the conclusion that long-term cultivated IT4^NE^ is a mixed population, in which most parasites contain the chromosomal deletion and are thus unable to form knobs, but a minority of the parasites do not contain the deletion. Binding or enrichment to CSA in the presence of increased temperature, therefore, appears to only be possible if the IEs have knobs, thereby selecting parasites without the deletion. Horrocks et al. demonstrated that parasites not expressing *kahrp* due to the chromosomal deletion display approximately half of the amount of *Pf*EMP1 on the IE surface and exhibit impaired cytoadhesion, which is consistent with the results of the present study [25].

Finally, we sought to determine if the selection of knobby parasites was restricted to CSA binding of IEs to HBEC-5i cells. As mentioned above, up to 23 ECRs have been identified as IE binding partners. Two of the most investigated receptors are ICAM-1 and CD36. ICAM-1 is a membrane-bound glycoprotein that has a central role in leukocyte trafficking, immunological synapse formation, and numerous cellular immune responses [56]. ICAM-1 is likely to be involved in malaria pathogenesis [57]. Unlike CSA, binding to ICAM-1 is mediated by multiple *Pf*EMP1s that contain DBLβ3 domains within the DC4-*Pf*EMP1 domain of group A proteins or the DBLβ5 domains of group B and C proteins, which are not present in VAR2CSA [5,33,58,59,60,61]. The enrichment process of IT4^NE^ parasites to transgenic CHO^ICAM-1^ cells at 40 °C also led to selection of knobby IEs. This indicates that the observed selection mechanisms also apply to other IE-receptor interactions.

Over 70% of IT4 *Pf*EMP1 proteins contain CIDRα2–6 domains, which are involved in CD36 binding [62]. Interestingly, the enrichment of IEs to CD36 at 40 °C did not lead to selection of knobby IEs. A prior study by Tilly et al. suggested that knobs are not as decisive for CD36 binding as for ICAM-1 binding. Tilly et al. demonstrated that CD36 binding capacity does not differ between knobless and knobby IEs. Knobby IEs, however, bind significantly better to ICAM-1 [63]. In this context, it can be postulated that binding to CD36, is so strong that accumulation of *Pf*EMP1 molecules within the knob structure is not necessary for CD36 binding under febrile temperatures.

Fever is the most apparent symptom during malaria infection. Reducing fever in children with malaria is controversial and creates therapeutic challenges [64]. On the one hand, fever increases the risk of seizures [65]. However, treatment with the antipyretic drug paracetamol slows parasite clearance due to decreased capacity for production of tumor necrosis factor (TNF) and oxygen radicals [66]. It has also been shown that febrile temperatures increase cytoadhesion [28,30], and the ability of IEs to adhere to certain microvascular endothelial cells correlates with the severity of malaria infection [67,68,69]. On the other hand, fever inhibits parasite growth and leads to parasite death [27,70].

## 5. Conclusions

The results of the present study reveal a previously unknown effect of febrile temperature during cytoadhesion. In the presence of febrile temperatures, binding to certain ECRs can only occur if the IEs have knobs. In addition, adhesion is enhanced by increased *var* expression and correspondingly increased IE surface P*f*EMP1. The increased cytoadhesion may, therefore, affect the clinical outcome of malaria infection. However, the mechanism that promotes binding of knobby IEs at febrile temperature is not known. One possibility is that febrile temperature causes conformational changes of *Pf*EMP1 molecules clustered in knobs and of the receptors, resulting in stronger binding. However, the mechanisms of enhanced adhesion at febrile temperatures requires further investigation. Despite this uncertainty, we were able to shed light on the complex interactions of the *Pf*EMP1-receptor with ECRs, and show how different environmental factors, such as febrile temperature, influence these interactions. In addition, our findings suggest that the selection pressure for knob formation in IEs originates from the host. Without knobs and the resultant improved cytoadhesion, parasitic survival would likely be impaired, particularly under febrile temperature.

## Figures and Tables

**Table 1:** Read counts of RNAseq analysis and normalized expression level determined using qPCR for selected genes of IT4^CSA^EC37°, IT4^CSA^EC40°, and IT4^NE^ heat-shocked at 38.5 °C and 40 °C (IT4^NE^+38.5 °C, IT4^NE^+40 °C) compared with IT4^NE^.

Gene Name	Gene ID	Read Counts (RNAseq) ^1^			Normalized Expression Level (qPCR)				Normalized Copy Number (gDNA, qPCR)	
		IT4^NE^	IT4^CSA^ EC37°	IT4^CSA^ EC40°	IT4^CSA^ EC37°	IT4^CSA^ EC40°	IT4^NE^ +38.5 °C	IT4^NE^ +40 °C	IT4^CSA^ EC37°	IT4^CSA^ EC40°
*var2csa*	PFIT_1200200	79	49285	94134	34	63	1.46	0.96	3.9	330
*IT4_var13*	PFIT_0411400	51	45	22	0.36	0.82	1.51	0.88	3.3	99
*IT4_var41*	PFIT_0900100	13	11	14	0.24	0.44	0.65	0.68		
*IT4_var02*	PFIT_bin08900	8	2	3	0.24	0.58	1.49	0.69		
*IT4_var28*	PFIT_0711000	100	57	74	0.15	0.38	1.32	0.35		
*kahrp*	PFIT_0201300	56	0	148657	10.23	3593.9	nd	nd		
*pfemp3*	PFIT_0201200	6	0	21077	3.14	453.9	nd	nd		

^1^Supplementary Table S2; nd: not determined.

**Table 2:** Upregulated genes (fold change ≥ 5) in ring-stage IT4^CSA^EC40° parasites compared to ring-stage IT4^CSA^EC37° parasites (padj < 0.01).

Gene Name	Gene ID	Read Counts (RNAseq)			
		IT4^CSA^EC37°	IT4^CSA^-EC40°	Fold Change	Reference
*knob-associated histidine-rich protein (kahrp)* * K	PFIT_0201300	0	148657	N/A	[41]
*erythrocyte membrane protein 3 (pfemp3)* * K	PFIT_0201200	0	21077	N/A	[42]
*knob associated heat shock protein 40 (kahsp40)* *K	PFIT_0201100	0	14136	N/A	[43]
*DnaJ protein* *	PFIT_0201000	0	4965	N/A	[44]
*PHISTb domain-containing RESA-like protein 1* * K	PFIT_0200900	0	3861	N/A	[44]
*rifin*	PFIT_0424300	0	2377	N/A	
*Plasmodium exported protein (hyp9)* *	PFIT_0200800	0	388	N/A	[45]
*rifin*	PFIT_bin10000	9	302	34	
*Plasmodium* exported protein (*phista*)	PFIT_0423600	7	108	16	
*ring-infected erythrocyte surface antigen 2*	PFIT_1149700	102	1405	14	
*Plasmodium* exported protein (*phistb*) **K**	PFIT_0423000	242	2535	10	[46]
*glycophorin binding protein* **K**	PFIT_1300400	272	2076	8	[43]
*Plasmodium exported protein (phista)*	PFIT_0423100	852	5709	7	
*asparagine-rich antigen, putative*	PFIT_0931800	25	158	6	
*Plasmodium exported protein*	PFIT_0725000	789	4099	5	
*acyl-CoA synthetase* **K**	PFIT_0801900	141	695	5	[46]
*virulence-associated protein 1*	PFIT_0937100	67	307	5	

* Located at the subtelomeric region of chromosome 2; **K** Associated with knob formation.

**Table 3:** Normalized expression level of various genes of IT4^ICAM-1^CHO^ICAM-1^40°, IT4^CD36^CHO^CD36^40° compared to IT4^NE.^determined using qPCR. For read counts (RNAseq) of IT4^NE^, IT4^NE^, IT4^ICAM-1^CHO^ICAM-1^37° and IT4^CD36^CHO^CD36^37° (* see [33]).

Gene Name	Gene ID	Normalized Expression Level (mRNA)		Normalized Expression Level (RNAseq) *		
		IT4^ICAM-1^ CHO^ICAM-1^40°	IT4^CD36^ CHO^CD36^40°	IT4^NE^	IT4^ICAM-1^ CHO^ICAM-1^37°	IT4^CD36^ CHO^CD36^37°
*kahrp*	PFIT_0201300	241	2	0	0.3	44
*pfemp3*	PFIT_0201200	56	1.3	0	0	2
*var01*	PFIT_0616500	107	1.6	68	55911	170
*var2csa*	PFIT_1200200	0.5	2.6	42	53	15
*var16*	PFIT_bin09100	15	1.7	22	3790	3

## Supplementary Materials

The following are available online at https://www.mdpi.com/2076-2607/8/2/174/s1.
