# Supplementary material for: Stringent Selection of Knobby Plasmodium falciparum-Infected Erythrocytes during Cytoadhesion at Febrile Temperature
Source: Microorganisms. 2020 Jan 25;8(2):174. doi: 10.3390/microorganisms8020174 (PMC7074740; doi:10.3390/microorganisms8020174)
Supplement: Supplementary file 1 [file microorganisms-08-00174-s001.zip › microorganisms-675149 supp - final check/Supplementary Figures S1 and S2.pdf]

SUPPLEMENTARY MATERIAL

Figure S1

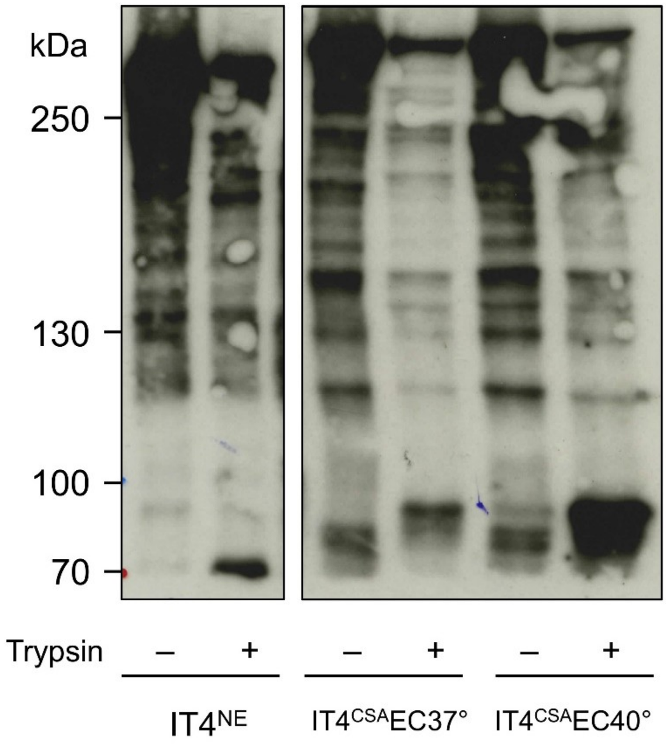

Figure S1: Western blot analysis for ATS was performed with trypsin-treated or untreated IEs and uninfected erythrocytes. All samples were solubilized in Laemmli sample buffer, separated by 6% SDS-PAGE, and analyzed by immunoblotting. Equivalents of 3 × 10^7 cells were loaded in each lane. The ATS antibody detects surface *Pf*EMP1 as trypsin-cleaved intracellular ATS-residues.

SUPPLEMENTARY MATERIAL

Figure S2

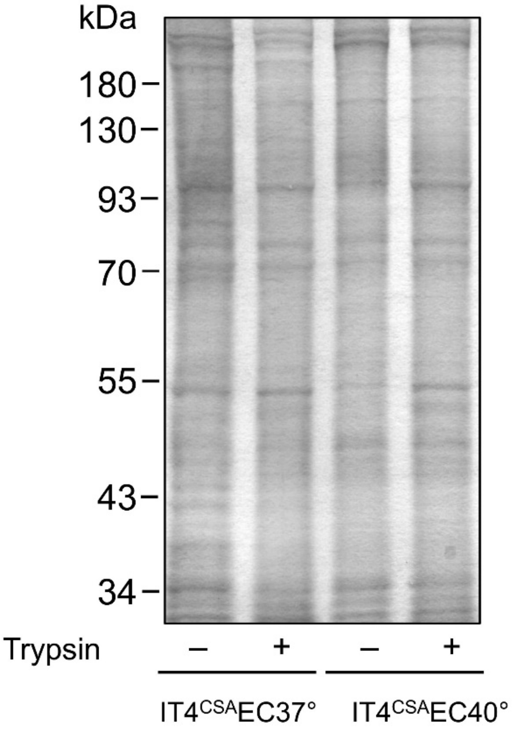

Figure S2: Coomassie-stained gel (10%) of membrane fractions (equivalents of 1×10^7 cells were loaded in each lane) of IT4^{CSA}EC37° and IT4^{CSA}EC40° parasite populations (control for Figure. 2A)
