# Supplementary material for: Stringent Selection of Knobby Plasmodium falciparum-Infected Erythrocytes during Cytoadhesion at Febrile Temperature
Source: Microorganisms. 2020 Jan 25;8(2):174. doi: 10.3390/microorganisms8020174 (PMC7074740; doi:10.3390/microorganisms8020174)
Supplement: Supplementary file 1 [file microorganisms-08-00174-s001.zip › microorganisms-675149 supp - final check/Supplementary Table S1 .docx]

**Supplementary Table S1:** Oligonucleotides used for qPCR analysis.

| ***Gene*** | **Gene ID** | **forward primer** | **reverse primer** |
| --- | --- | --- | --- |
| *IT4_var01* | PFIT_0616500 | GCCCACCTAGGAACATATAAAC | TCACCGTCAGTACGACTATC |
| *IT4_var02* | PFIT_bin08900 | GGGAAAGACAACGACAAAGA | ACGAGGAGGTGTCGATTTA |
| *IT4_var04 (var2csa)* | PFIT_1200200 | GGTAAAGGAGGCGAGAAAC | CCCATAACTCTCCAACACATAA |
| *IT4_var13* | PFIT_0411400 | AAACCGTAAGCCACAAGAG | GTACATGCTCCACCGTTATT |
| *IT4_var16* | PFIT_bin09100 | CCAATCGTAATCCTGCTCAA | CTTACCTGTTCTCCACCTTTC |
| *IT4_var28* | PFIT_0711000 | TTCGTGACGCCGATAGT | GTCGAAACCACCAAGGTATAG |
| *IT4_var41* | PFIT_0900100 | CCAATCGTAATCCTGCTCAA | CTTACCTGTTCTCCACCTTTC |
| *kahrp* | PFIT_0201300 | CCATCATCACCACCATCATC | GGTCTTGCTTCCCTGAATAC |
| *pfemp3* | PFIT_0201200 | AATCAGCAGGTCATCCATTT | GGTTGGTGGTTCATGTTCT |
| *fructose-bisphosphate aldolase* | [PFIT_1446000](http://plasmodb.org/plasmo/app/record/gene/PFIT_1446000) | TGTACCACCAGCCTTACCAG | TTCCTTGCCATGTGTTCAAT |
| *skeleton-binding protein 1 (sbp1)* | [PFIT_0501400](http://plasmodb.org/plasmo/app/record/gene/PFIT_0501400) | TTAGCCGACGAACCAACACA | TTCGGTTGTCTCTGGTACTGCA |
| *arginyl-tRNA-synthetase* | [PFIT_1218300](http://plasmodb.org/plasmo/app/record/gene/PFIT_1218300) | TTCAAAACACGAAGTGGAACAAC | AATTCTCTGCAGCAAGTCGC |
